# Ureteropelvic junction obstruction caused by metastatic cholangiocarcinoma

**DOI:** 10.1590/S1677-5538.IBJU.2019.0053

**Published:** 2019-12-17

**Authors:** Nicholas A. Pickersgill, Alec J. Wright, Robert S. Figenshau

**Affiliations:** 1 Department of Surgery, Division of Urologic Surgery, Washington University School of Medicine, St. Louis, MO, USA;; 2 Mallinckrodt Institute of Radiology, Washington University School of Medicine, St. Louis, MO, USA

**Keywords:** Ureteropelvic junction obstruction, neoplasm, metastatic, pyeloplasty, cholangiocarcinoma

## Abstract

We describe the rare case of a 61-year-old female with right ureteropelvic junction (UPJ) obstruction caused by metastatic cholangiocarcinoma. Her past medical history was notable for cholangiocarcinoma treated with neoadjuvant chemoradiation and two orthotopic liver transplants six years earlier. Urology was consulted when she presented with flank pain and urinary tract infection. Diagnostic workup demonstrated right UPJ obstruction. She was managed acutely with percutaneous nephrostomy. She subsequently underwent robotic pyeloplasty and intrinsic obstruction of the UPJ was discovered. Histological examination revealed adenocarcinoma, consistent with systemic recurrence of the patient's known cholangiocarcinoma.

## INTRODUCTION

Obstruction of the ureteropelvic junction (UPJ) is a common urological problem that can result in hydronephrosis and deterioration of renal function. Although most commonly observed in pediatric populations, UPJ obstruction in adults can arise from a number of etiologies, including congenital causes, acquired stenosis due to urolithiasis, urothelial malignancy, and other retroperitoneal disease processes (1, 2). Intraluminal malignant obstruction due to metastatic involvement of the UPJ, however, is exceedingly rare, with few case reports existing in the literature (3, 4). Herein we describe the case of a patient with a history of hilar cholangiocarcinoma treated with a liver transplant, whose systemic recurrence presented as an isolated right UPJ obstruction six years later.

## CASE REPORT

**Figure 1:**
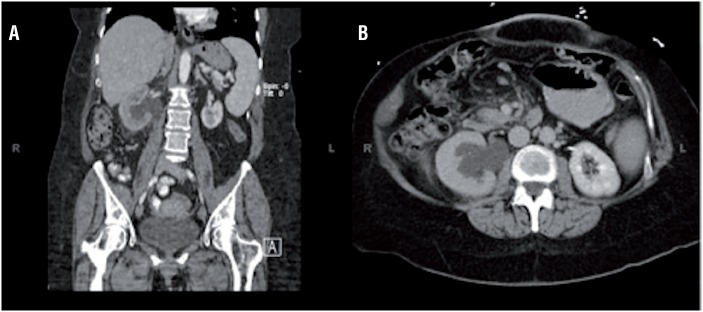
Coronal (a) and axial (b) contrast-enhanced computed tomography revealed moderate-to-severe right hydronephrosis with decreased nephrogram of the right kidney, concerning for high-grade right UPJ obstruction.

A 61-year-old female presented to the emergency department with right flank pain, fever, chills, nausea, and vomiting. Urinalysis showed positive nitrites, moderate leukocyte esterase, 4+ bacteria, and >100 white blood cells on microscopy. An abdominal/pelvic CT scan was obtained which revealed moderate-to-severe right hydronephrosis with delayed right nephrogram, concerning for high-grade right UPJ obstruction (Figure-1). No associated mass, lymphadenopathy, calculus, or other cause of obstruction was identified. Her past medical history was notable for hilar cholangiocarcinoma treated with neoadjuvant chemoradiation followed by two orthotopic liver transplants six years prior with no interval evidence of recurrence or metastasis.

**Figure 2:**
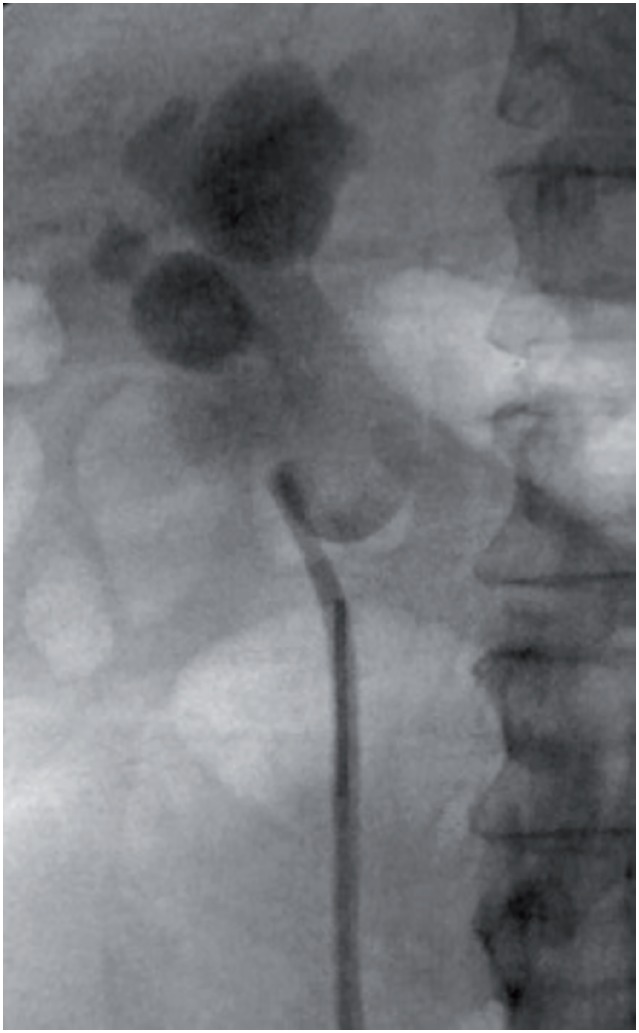
Right retrograde pyelogram performed prior to stent placement revealed moderate hydronephrosis and features concerning for ureteropelvic junction obstruction.

The patient was admitted to the hospital and treated with intravenous antibiotics for acute obstructive pyelonephritis. A retrograde pyelogram was performed which revealed a dilated collecting system and features concerning for UPJ configuration (Figure-2), and a right ureteral stent was placed under fluoroscopy prior to discharge.

One month later, the patient presented again to the emergency department with continued right flank pain. CT abdomen/pelvis revealed persistent hydronephrosis, necessitating placement of a right percutaneous nephrostomy tube. During subsequent follow-up, diuretic renal scintigraphy performed with the nephrostomy tube clamped demonstrated persistent high-grade right-sided obstruction. The patient was counseled on treatment options and elected to undergo a right retroperitoneal robotic-assisted laparoscopic pyeloplasty. At this time, there was no suspicion of malignant obstruction based upon preoperative imaging, and the UPJ obstruction was presumed to have been either congenital or due to retroperitoneal fibrosis caused by the patient's previous liver transplants.

Intraoperatively, dissection of the lower pole of the right kidney revealed a moderate degree of fibrosis. Amidst the fibrosis, the ureter was identified and followed cephalad to the dilated renal pelvis. The UPJ was dissected and the renal pelvis was opened, revealing an obstructive intrinsic papillary lesion at the UPJ, which was biopsied and sent for frozen section. Histopathological analysis of the frozen section revealed findings concerning for adenocarcinoma. The UPJ area was then resected back to what appeared to be uninvolved mucosa. A dismembered pyeloplasty was performed and a right ureteral stent was placed.

**Figure 3:**
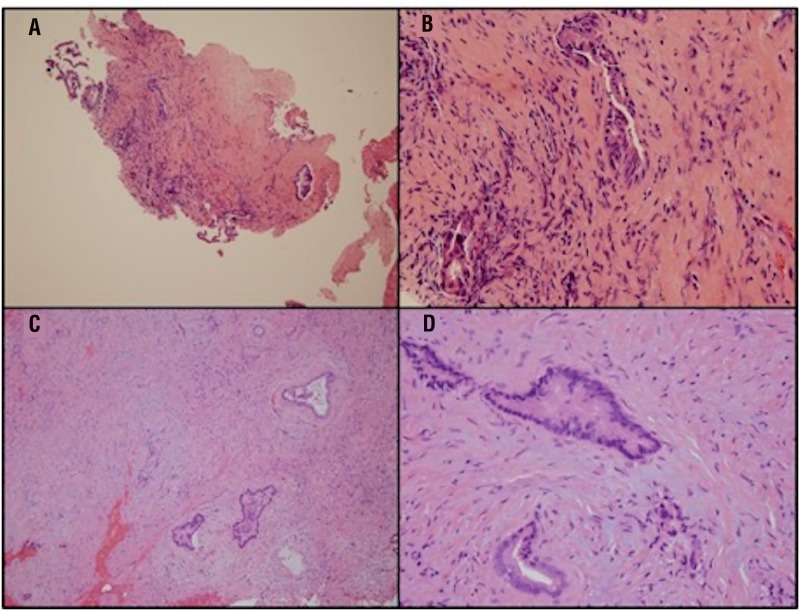
Invasive moderate-to poorly-differentiated adenocarcinoma infiltrating bile duct fibrotic stroma seen in 100x (a) and 400x (b) magnifications. Right ureteropelvic junction biopsy is shown in 100x (c) and 400x (d) magnifications. Similarly, relatively bland low magnification morphology is seen in the primary (a) and metastatic (c) cholangiocarcinoma. Hyperchromasia and high nuclear-to-cytoplasmic ratios are better appreciated at higher magnification. Desmoplastic stromal response is seen around infiltrating malignant glands (d).

The UPJ and periureteral fibrotic tissue were sent for permanent section. Histopathologic sections of all biopsy specimens revealed infiltrating, atypical glands lined by columnar tumor cells with nuclear and cytoplasmic abnormalities (Figure-3). The morphological consistency with the patient's known cholangiocarcinoma, combined with the patient's history, were concerning for a metastatic cholangiocarcinoma recurrence at the UPJ.

Upon histopathologic diagnosis of her ureteral metastases, the patient was started on a regimen of gemcitabine and cisplatin, which is currently considered the reference regimen for advanced biliary cancer (5). Both medications were subsequently discontinued after five months due to fatigue and poor patient tolerance. Her right UPJ obstruction has persisted since surgery, presumably due to persistent cholangiocarcinoma within the retroperitoneum observed on surveillance CT scans. This has been managed with percutaneous nephrostomy drainage as she has not tolerated ureteral stents. Interestingly, at 1-year, there remains no radiographic evidence of disease progression.

## DISCUSSION

While malignancies of the genitourinary system are common, it is estimated that only 1.6%-3.0% represent secondary tumors (6). Of these, most present either in the kidney or bladder. There have been many reported cases of metastatic spread from the pancreas, stomach, breast, prostate, lung, and colon presenting initially as ureteral obstruction (7–13). The vast majority of these cases present as extrinsic obstruction secondary to retroperitoneal lymphadenopathy. Secondary malignancies causing intrinsic blockage and presenting as isolated UPJ obstructions, on the other hand, are extremely rare in the literature. Naranji et al. (4) reported a case of right-sided UPJ obstruction in a 78-year-old woman who underwent laparoscopic pyeloplasty that showed mantle cell lymphoma on final histology. The only reported observation of malignant infiltration of the UPJ by a solid organ cancer is a case of recurrent breast cancer reported by Shah et al. (3) in 2016. Importantly, to the best of our knowledge, this case report is the first to describe metastasis of a primary biliary tract malignancy to the UPJ.

## CONCLUSION

We describe a rare presentation of metastatic cholangiocarcinoma causing intrinsic UPJ obstruction. In patients with a history of malignancy, physicians should consider metastatic relapse in the differential diagnosis for acquired UPJ obstruction. Endoscopic evaluation may be helpful in both diagnosis and the planning of surgical intervention.

## References

[1] 1. XGrasso M, Caruso RP, Phillips CK. UPJ Obstruction in the Adult Population: Are Crossing Vessels Significant? Rev Urol. 2001;3:42-51. PMC1476031 16985690

[2] 2. Park JM, Bloom DA. The pathophysiology of UPJ obstruction. Current concepts. Urol Clin North Am. 1998;25:161-9. 10.1016/s0094-0143(05)70004-5 9633571

[3] 3. Shah PH, Smith AT, Leavitt DA, Yaskiv O, Kavoussi LR. Ureteropelvic Junction Obstruction Secondary to Metastatic Relapse of Breast Cancer. Urol Case Rep. 2015;4:38-40. 10.1016/j.eucr.2015.08.004 PMC4719903 26793576

[4] 4. Naranji I, Zakri RH, Liston T. Mantle cell lymphoma presenting as a pelvi-ureteric junction obstruction: a case report. J Med Case Rep. 2013;7:105. 10.1186/1752-1947-7-105 PMC3637467 23590763

[5] 5. Valle J, Wasan H, Palmer DH, Cunningham D, Anthoney A, Maraveyas A, ET AL. Cisplatin plus gemcitabine versus gemcitabine for biliary tract cancer. N Engl J Med. 2010;362:1273-81. 10.1056/NEJMoa0908721 20375404

[6] 6. Bates AW, Baithun SI. The significance of secondary neoplasms of the urinary and male genital tract. Virchows Arch. 2002;440:640-7. 10.1007/s00428-001-0549-x 12070605

[7] 7. Nikolaos F, Panagiotis A, Konstantinos B, Vassilios S, Iraklis P. Distant ureteral metastasis from colon adenocarcinoma: report of a case and review of the literature. Case Rep Urol. 2014;2014:196425. 10.1155/2014/196425 PMC3971491 24716082

[8] 8. Schallier D, Rappe B, Carprieaux M, Vandenbroucke F Ureteral Metastasis: Uncommon Manifestation in Prostate Cancer. Anticancer Res. 2015;35:6317-20. 26504069

[9] 9. Schneider S, Popp D, Denzinger S, et al. A rare location of metastasis from prostate cancer: Hydronephrosis associated with ureteral metastasis. Adv Urol. 2012 [cited Jul 27, 2018]; [3 p.]. 10.1155/2012/656023 PMC3168270 21912541

[10] 10. Zhang D, Li H, Gan W. Hydronephrosis associated with ureteral metastasis of prostate cancer: A rare case report. Mol Clin Oncol. 2016;4:597-598. 10.3892/mco.2016.775 PMC4812183 27073671

[11] 11. Bisof V, Juretic A, Pasini J, Coric M, Grgic M, Gamulin M, et al. Ureteral metastasis as the first and sole manifestation of gastric cancer dissemination. Radiol Oncol. 2010;44:262-4. 10.2478/v10019-010-0015-y PMC3423711 22933926

[12] 12. Arvind NK, Singh O, Gupta S, Ali Q. Ureteral metastasis as the presenting manifestation of pancreatic carcinoma. Rev Urol. 2013;15:124-30. PMC3821992 24223025

[13] 13. Huang TB, Ding PP, Chen JF, Yan Y, Zhang L, Liu H, et al. Dietary fiber intake and risk of renal cell carcinoma: evidence from a meta-analysis. Med Oncol. 2014;31:125. 10.1007/s12032-014-0125-2 25038944

